# Glucocorticoids Regulation of FosB/ΔFosB Expression Induced by Chronic Opiate Exposure in the Brain Stress System

**DOI:** 10.1371/journal.pone.0050264

**Published:** 2012-11-21

**Authors:** Daniel García-Pérez, M. Luisa Laorden, M. Victoria Milanés, Cristina Núñez

**Affiliations:** Group of Cellular and Molecular Pharmacology, Department of Pharmacology, University School of Medicine, Murcia, Spain; University of California, Los Angeles, United States of America

## Abstract

Chronic use of drugs of abuse profoundly alters stress-responsive system. Repeated exposure to morphine leads to accumulation of the transcription factor ΔFosB, particularly in brain areas associated with reward and stress. The persistent effects of ΔFosB on target genes may play an important role in the plasticity induced by drugs of abuse. Recent evidence suggests that stress-related hormones (e.g., glucocorticoids, GC) may induce adaptations in the brain stress system that is likely to involve alteration in gene expression and transcription factors. This study examined the role of GC in regulation of FosB/ΔFosB in both hypothalamic and extrahypothalamic brain stress systems during morphine dependence. For that, expression of FosB/ΔFosB was measured in control (sham-operated) and adrenalectomized (ADX) rats that were made opiate dependent after ten days of morphine treatment. In sham-operated rats, FosB/ΔFosB was induced after chronic morphine administration in all the brain stress areas investigated: nucleus accumbens(shell) (NAc), bed nucleus of the stria terminalis (BNST), central amygdala (CeA), hypothalamic paraventricular nucleus (PVN) and nucleus of the solitary tract noradrenergic cell group (NTS-A_2_). Adrenalectomy attenuated the increased production of FosB/ΔFosB observed after chronic morphine exposure in NAc, CeA, and NTS. Furthermore, ADX decreased expression of FosB/ΔFosB within CRH-positive neurons of the BNST, PVN and CeA. Similar results were obtained in NTS-A_2_ TH-positive neurons and NAc pro-dynorphin-positive neurons. These data suggest that neuroadaptation (estimated as accumulation of FosB/ΔFosB) to opiates in brain areas associated with stress is modulated by GC, supporting the evidence of a link between brain stress hormones and addiction.

## Introduction

Opiate drugs, such as morphine, are effective analgesic agents that are used for treating many forms of acute and chronic pain. However, serious adverse effects such as tolerance and withdrawal contribute to opiate dependence and limit their use. Further, the non-medical use of opiates (heroin, morphine) has increased during the past few years. Increasing evidence implicates various mechanisms of gene regulation (including epigenetic, molecular, cellular and circuit level effects) in the changes that drugs of abuse induce in the brain, indicating a potential therapeutic strategy for addiction therapy [1]–[4].

A central question in the drug abuse field has been to identify proteins that mediate the transition from acute to long-term effects of those drugs. Of particular interest in the study of addiction is the Fos family of transcription factors. This family includes c-Fos, Fra-1 and Fra-2, FosB and ΔFosB, a truncated splice variant of full-length FosB [5]. In contrast to other members of the Fos family, ΔFosB is modestly induced in the brain after acute drug administration, but because of its unusual long half-life it persists for weeks, even months, after the cessation of drug use. As a result, ΔFosB levels gradually accumulate with repeated drug exposure [6], [7], suggesting that ΔFosB could represent a mechanism by which drugs of abuse produce lasting changes in gene expression pattern long after the drug is withdrawn [8].

It has been reported that repeated exposure to cocaine, amphetamine, cannabinoids or morphine leads to an increase in ΔFosB in brain areas related to the positive reinforcing effects of drugs, such as nucleus accumbens (NAc), prefrontal cortex and dorsal striatum. This increase has been proposed to be a neuroadaptation that leads to increased sensitivity to drugs of abuse and vulnerability to develop characteristic behaviours of addiction [9]–[13]. We have recently shown that the enhancement of FosB/ΔFosB levels after chronic morphine administration is not only restricted to the reward system, but also occurs in the brain stress system (which has been related to the negative reinforcing effects of drugs), as well as in the nucleus of the solitary tract-A_2_ noradrenergic cell group (NTS-A_2_, the main noradrenergic system innervating the stress neurocircuitry) [14]. In consonance with these findings, several forms of chronic stress also induce ΔFosB in the NAc and other brain regions [15], [16].

Addiction is a complex disorder because many factors contribute to the development and maintenance of this neurological disorder. One factor is stress, which has been involved in specific aspects of drug addiction [17]–[21]. Both the hypothalamus-pituitary-adrenal (HPA, the primary endocrine stress pathway) axis and the extrahypothalamic stress system (which comprises the extended amygdala and the NTS-A_2_) are dysregulated by chronic administration of drugs with dependence potential [14], [22]. In addition, HPA axis response is similar after both stressful stimuli and acute exposure to drugs of abuse [23], [24], with elevated corticotropin-releasing hormone (CRH), adrenocorticotropic hormone (ACTH), and glucocorticoids (GC) release. This response facilitates adaptation to acute environmental changes, but also may lead to behavioural pathologies during chronic stress conditions, such as addiction and depression [25]. Studies have not yet examined the relationship between GC and morphine dependence-induced FosB/ΔFosB induction in the brain stress system. We therefore assessed the influence of GC on FosB/ΔFosB expression after continuous administration of morphine in brain stress-related areas. To address this question, we first examined the effects of bilateral adrenalectomy (ADX) on FosB/ΔFosB immunoreactivity (IR) in the main nuclei of the stress system in morphine-dependent rats.

The activity of the brain stress system is mediated by a number of neurotransmitters/neuromodulators. CRH is the main neuropeptide regulating the stress system activity and it has been postulated its contribution in both a pre‐existing vulnerability to use drugs addictively and later vulnerability to relapse [26]. In addition, abundant work supports the importance of the NTS-A_2_ innervating the brain stress system in drug addiction and the pivotal role of noradrenaline (NA) as a neurotransmitter modulating this neurocircuitry [27]. Finally, substantial evidence suggests that dynorphin expression is activated in the striatum and amygdala during acute and chronic drug administration [28]. Given these facts, the following aim of this study was to identify the role of GC on FosB/ΔFosB expression in specific populations of the brain stress system during morphine dependence.

## Results

### Effects of Adrenalectomy on Body Weight Gain and Plasma ACTH and Corticosterone Concentration in Morphine-dependent Rats

Before performing the immunodetection assays, we assessed the efficacy of chronic treatment with morphine. For this purpose, the weight of animals was recorded on the day of pellets implantation and on the day of killing (day 10). Two-way ANOVA revealed significant main effects on body weight gain for adrenalectomy [F(1,47) = 13.24, p* = *0.0007), morphine treatment [F(1,47) = 281.05, p*<*0.0001] and an interaction between ADX and morphine treatment [F(1,47) = 4.13, p* = *0.0479]. In accordance with previous findings [29], [30], *post hoc* analysis indicated that both sham and ADX groups rendered dependent on morphine showed a significantly (p*<*0.001) lower weight gain (−13.75±5.0 g, n = 12; 3.84±2.45 g, n = 13, respectively) than that observed in sham and ADX animals receiving placebo pellets (44.58±1.7 g, n = 12; 49.57±2.4 g, n = 12, respectively), which has been attributed to the reduced food intake observed in these animals [29].

To check the efficacy of the adrenalectomy, hormone concentrations were measured in plasma. Two-way ANOVA examining effects of adrenalectomy and morphine on ACTH and corticosterone plasma concentration showed significant main effects of adrenalectomy [ACTH: F(1,18) = 68.12, p*<*0.0001; corticosterone: F(1,45) = 10.42, p* = *0.0023). As expected, Newman’s *post hoc* test showed (Fig. S1) that in placebo (n = 6)- and morphine (n = 4)-ADX rats plasma concentration of ACTH was higher (p*<*0.001) compared with sham-operated animals for the two treatment evaluated (placebo, n = 6; and morphine, n = 6). No modifications were observed in plasma corticosterone concentration between ADX-placebo (n = 14) and ADX-morphine (n = 13) treated rats. Concentration of corticosterone in ADX-morphine treated rats were significantly (p*<*0.01) lower than those seen in sham-morphine (n = 10) treated rats. No significant changes were seen in sham-morphine group compared with sham-placebo (n = 12).

### Adrenalectomy Differentially Attenuates Morphine Dependence-induced FosB/ΔFosB in the Brain Stress System Sub Regions

In control animals (placebo-implanted rats), weak constitutive expression of FosB/ΔFosB-IR was identified in all the areas of the stress-related brain system. Chronic morphine treatment resulted in the appearance of FosB/ΔFosB in all the brain areas investigated. In the NAc(shell), two-way ANOVA showed significant main effects of adrenalectomy [F(1,16) = 6.44, p* = *0.0220] and chronic morphine treatments [F(1,16) = 19.98, p* = *0.0004]. Newman-Keuls’ post hoc test showed (Fig. 2A–E) that in morphine-treated rats there was a significant (p<0.01) increase in FosB/ΔFosB expression. In morphine-dependent ADX rats, there was a significant (p<0.05) reduction in FosB/ΔFosB protein expression in the NAc. Two-way ANOVA for BNST showed significant main effect of chronic morphine treatment [F(1,16) = 48.92, p*<*0.0001]. Post hoc analysis revealed an increase (p*<*0.001) in FosB/ΔFosB in both sham- and ADX-morphine dependent animals, indicating that regulation of FosB/ΔFosB was related to chronic morphine exposure irrespective of GC concentration (Fig. 2F–J). Two-way ANOVA for FosB/ΔFosB in the CeA showed significant effects of adrenalectomy [F(1,15) = 20.51, p* = *0.0004] and morphine pretreatment [F(1,15) = 27.52, p*<*0.0001]. *Post hoc* test showed a significant increase (p<0.01) in FosB/ΔFosB levels in sham-morphine rats versus sham-placebo rats, which was attenuated (p<0.01) in the ADX morphine-dependent group (Fig. 2K–O). In addition, ADX placebo-pelleted rats showed lower levels (p<0.01) of FosB/ΔFosB than the sham placebo group.

**Figure 1:**
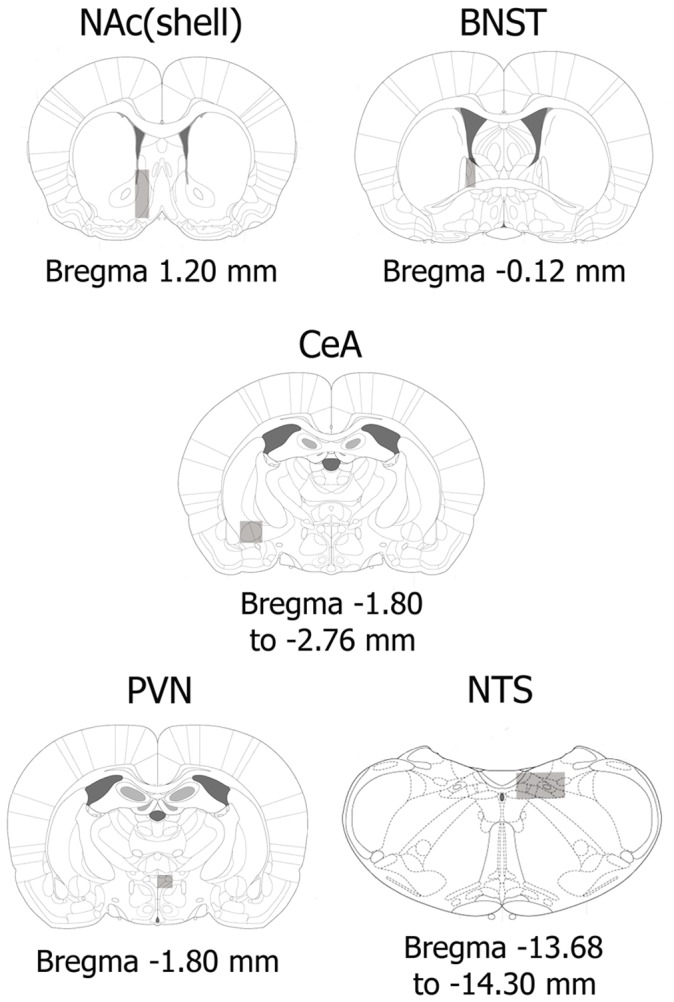
Schematic drawings of coronal sections indicating brain areas (rectangles) in which FosB/ΔFosB positive nuclei were counted in the NAc(shell), BNST, CeA, PVN and NTS [**59**].

**Figure 2:**
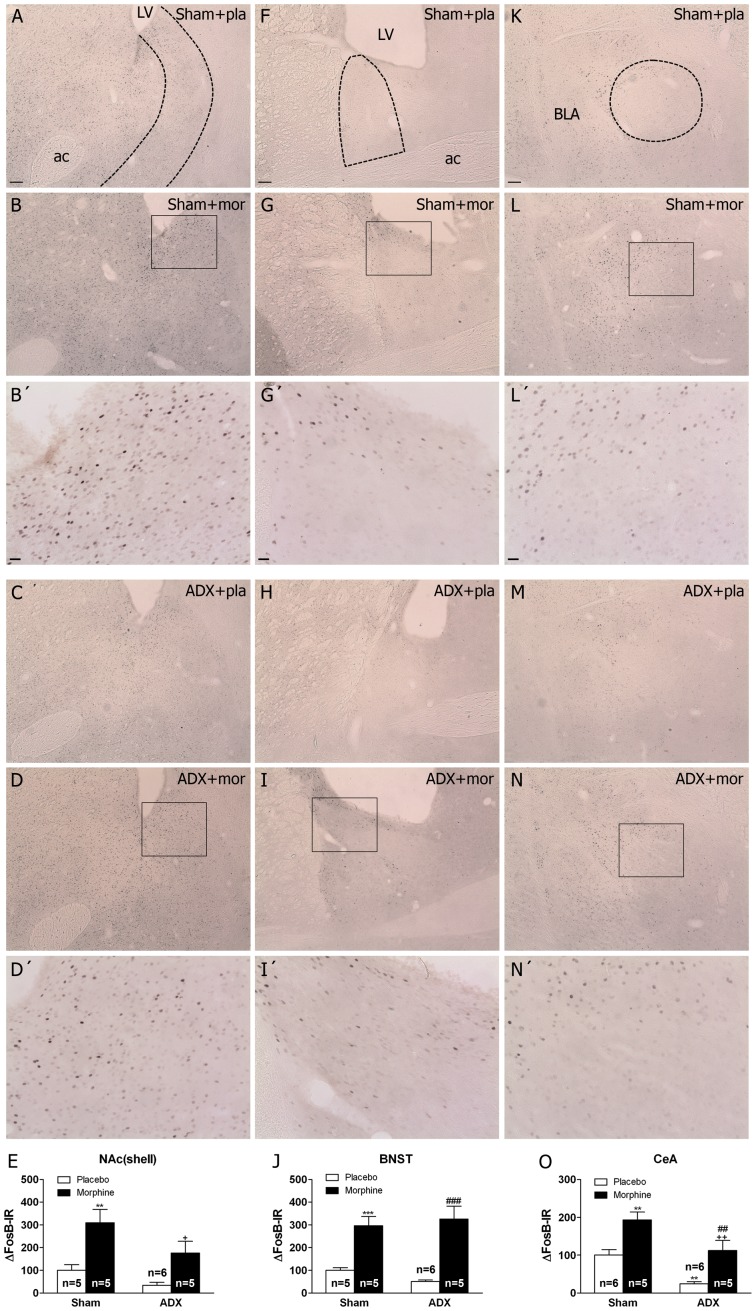
Adrenalectomy differentially regulates FosB/ΔFosB protein expression in the brain stress systems. Photographs represent immunohistochemical detection of FosB/ΔFosB in the NAc(shell) (A-D), BNST (F-I) and CeA (K-N) from sham-operated and adrenalectomized (ADX) rats pretreated with placebo (pla) or morphine (mor) pellets for 10 days. B’, D’. G’, I’, L’ and N’ are high magnifications. Scale bar: 100 µm (30X, low magnification); 20 µm (100X, high magnification). LV, lateral ventricle; ac, anterior comisure; BLA, basolateral amygdala. E, J, O, quantitative analysis of FosB/ΔFosB immunoreactivity in the three nuclei from sham and ADX animal. Data correspond to the mean ± SEM (percent of control). *Post hoc* comparisons revealed a significant increase of FosB/ΔFosB protein expression in sham animals after chronic morphine exposure in the NAc(shell), BNST and CeA (**p<0.01; ***p*<*0.001 versus sham-placebo animals). In ADX-morphine dependent rats it was observed an attenuation of FosB/ΔFosB expression in the NAc(shell) and CeA compared with sham-dependent rats (^+^p*<*0.05; ^++^p*<*0.01 versus sham+morphine). In addition, ADX decreased basal FosB/ΔFosB expression in the CeA (^**^p*<*0.01 versus sham-placebo group. ^##^p*<*0.01; ^###^p*<*0.001 versus ADX+placebo).

**Figure 3:**
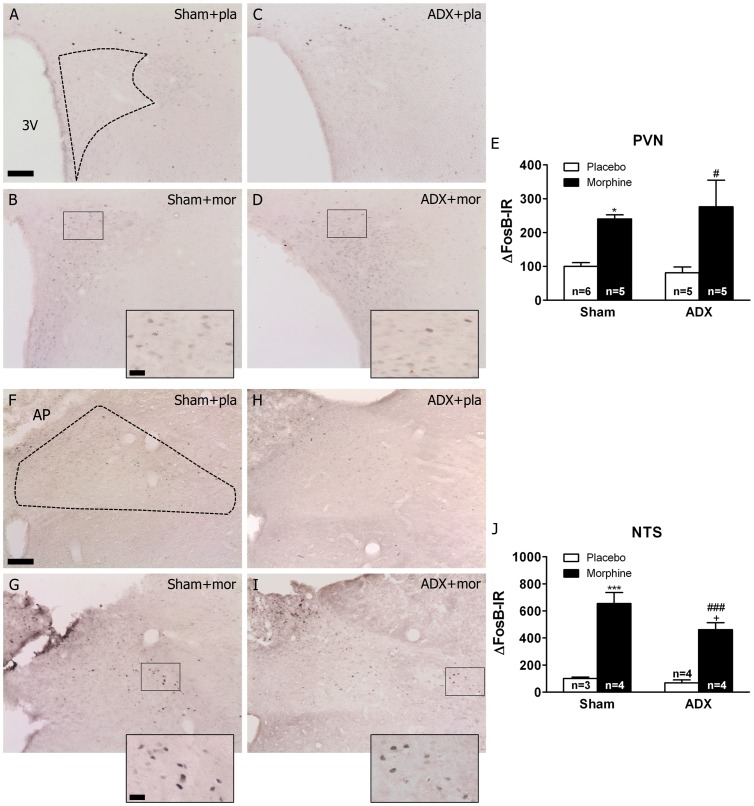
Effects of adrenalectomy on FosB/ΔFosB protein expression in the PVN and NTS-A_2_ from morphine-dependent rats. Photographs represent immunohistochemical detection of FosB/ΔFosB in the PVN (A-D), and NTS (F-I) neurons from sham-operated and ADX rats pretreated with placebo (pla) or morphine (mor) pellets for 10 days. Scale bar: 100 µm (70X, low magnification); 20 µm (200X, high magnification). 3 V, third ventricle; AP, area postrema; CC, canal central. Data represent the mean ± SEM (percent of control). E, J: quantitative analysis of FosB/ΔFosB-IR in the PVN and NTS, respectively. *p*<*0.05 ***p*<*0.001 versus sham+placebo; ^#^p*<*0.05, ^###^p*<*0.001 versus ADX+placebo; ^+^p<0.05 versus sham+morphine.

Two-way ANOVA examining the effects of adrenalectomy and morphine on FosB/ΔFosB expression in the PVN (parvocellular subdivision) revealed significant effect of morphine treatment [F(1,16) = 16.31, p*<*0.0001]. *Post hoc* analysis showed a significant (Fig. 3A–E) increase (p*<*0.05) in FosB/ΔFosB-IR in the PVN from both sham- and ADX-morphine treated rats. In the NTS-A_2_ catecholaminergic cell group, two-way ANOVA revealed a main effect of morphine treatment [F(1,11) = 76.33, p*<*0.0001]. *Post hoc* analysis showed a significant (Fig. 3F–J) increase in FosB/ΔFosB-IR in the sham-morphine rats compared with the sham-placebo group (p*<*0.001), which was attenuated (p<0.05) in the ADX morphine-dependent group.

### Chronic Morphine Exposure Induces FosB/ΔFosB Expression into CRH Neurons in PVN, BNST and CeA. Influence of Glucocorticoids

**Figure 4:**
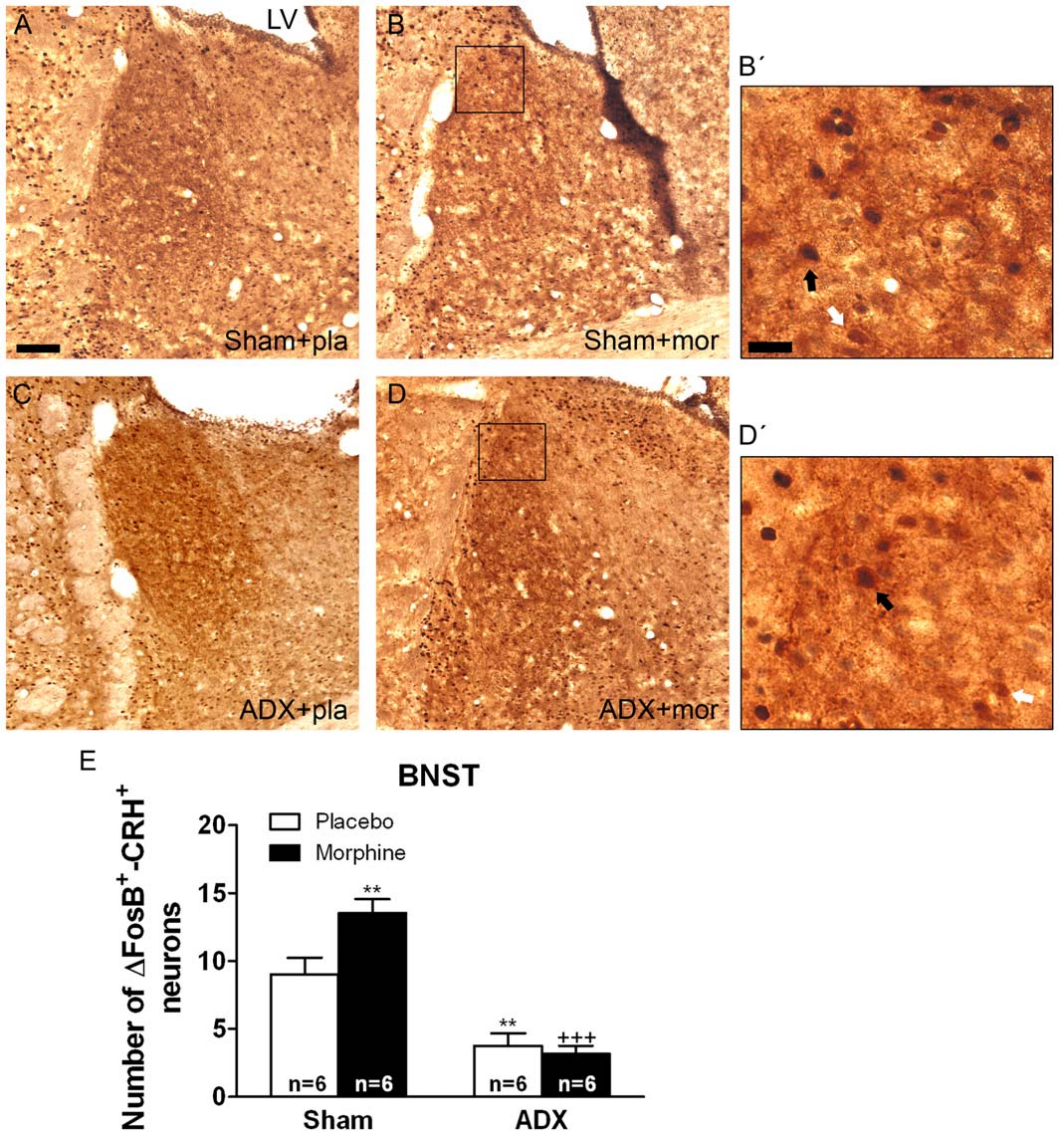
Adrenalectomy attenuated FosB/ΔFosB protein expression in the BNST CRH-positive neurons. ADX and non-operated (sham) rats were made dependent on morphine (mor) for 10 days. Controls received placebo pellets (pla). Animals were perfused and the BNST was processed for double-labelled (FosB/ΔFosB and CRH) immunohistochemistry. Panels A-D show immunohistochemical detection of FosB/ΔFosB into CRH neurons after different treatments. Low and high magnifications (B’, D’) images show FosB/ΔFosB-positive (blue-black)/CRH-positive (brown) neurons (black arrow) and FosB/ΔFosB-negative/CRH-positive (white arrow) immunoreactivity. Scale bar: 100 µm (50X, low magnification); 20 µm (250X, high magnification). LV, lateral ventricle. E: quantitative analysis of FosB/ΔFosB-positive/CRH-positive neurons in the BNST. Data correspond to mean ± SEM. *Post hoc* test revealed a significant higher number of FosB/ΔFosB-positive nuclei into CRH immunoreactive neurons in morphine-dependent rats (**p<0.01 versus sham+placebo). ADX attenuated the increase of FosB/ΔFosB expression in CRH-positive neurons both in placebo- (**p<0.01 versus sham+placebo) and in morphine-treated animals (^+++^p<0.001 versus sham+morphine).

**Figure 5:**
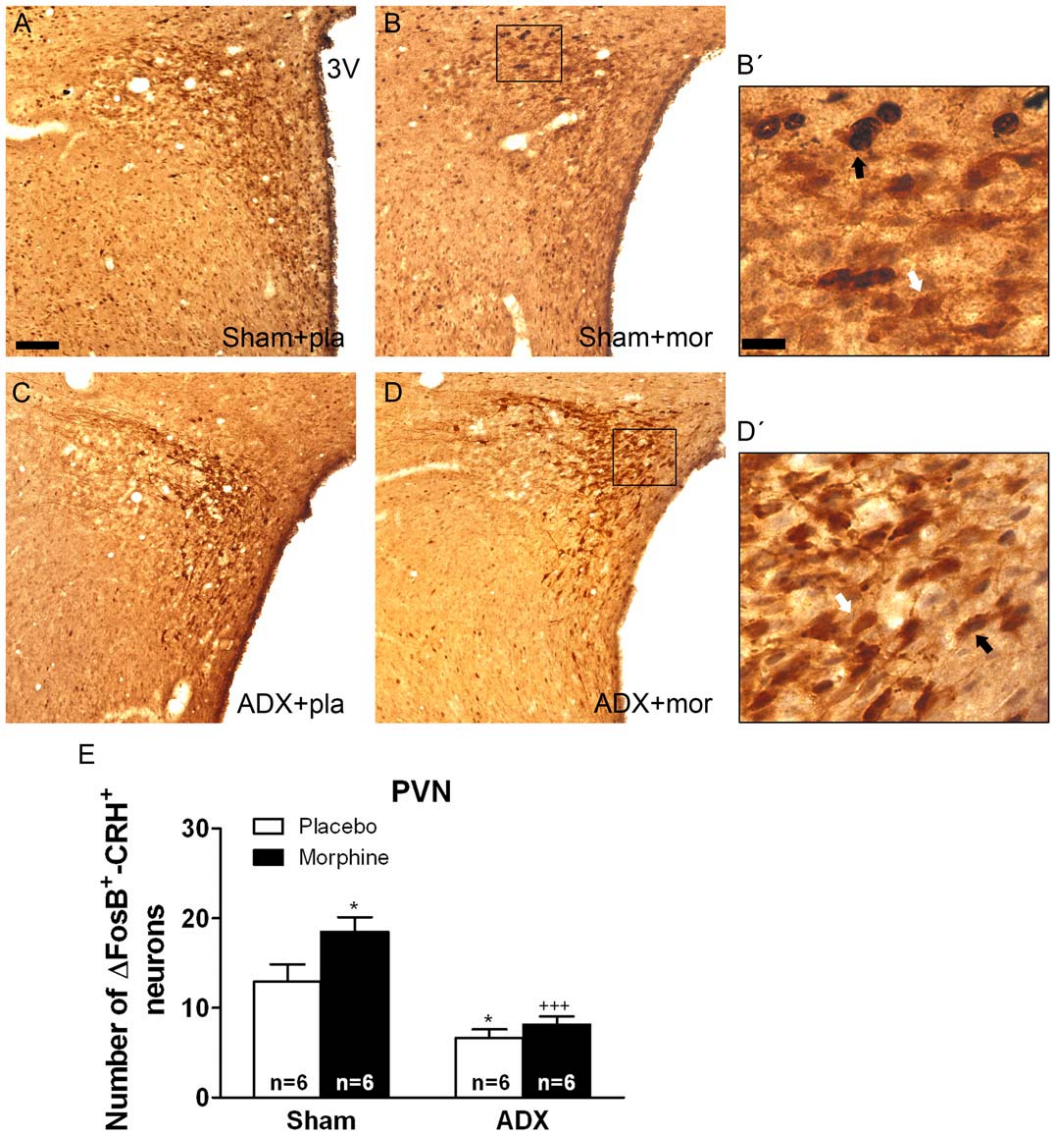
Adrenalectomy attenuated FosB/ΔFosB protein expression in the PVN CRH-positive neurons. ADX and non-operated (sham) rats were made dependent on morphine (mor) for 10 days. Controls received placebo pellets (pla). Animals were perfused and the PVN was processed for double-labelled (FosB/ΔFosB and CRH) immunohistochemistry. Panels A-D show immunohistochemical detection of FosB/ΔFosB into CRH neurons after different treatments. Low and high magnifications (B’, D’) images show FosB/ΔFosB-positive (blue-black)/CRH-positive (brown) neurons (black arrow) and FosB/ΔFosB-negative/CRH-positive (white arrow) immunoreactivity. Scale bar: 100 µm (50X, low magnification); 20 µm (250X, high magnification). 3 V, third ventricle. E: quantitative analysis of FosB/ΔFosB-positive/CRH-positive neurons in the PVN. Data correspond to mean ± SEM. *Post hoc* test revealed a significant higher number of FosB/ΔFosB-positive nuclei into CRH immunoreactive neurons in morphine-dependent rats (*p<0.05 versus sham-placebo). ADX attenuated the increase of FosB/ΔFosB expression in CRH-positive neurons both in placebo- (*p<0.01 versus sham+placebo) and in morphine-treated animals (^+++^p*<*0.001 versus sham+morphine).

**Figure 6:**
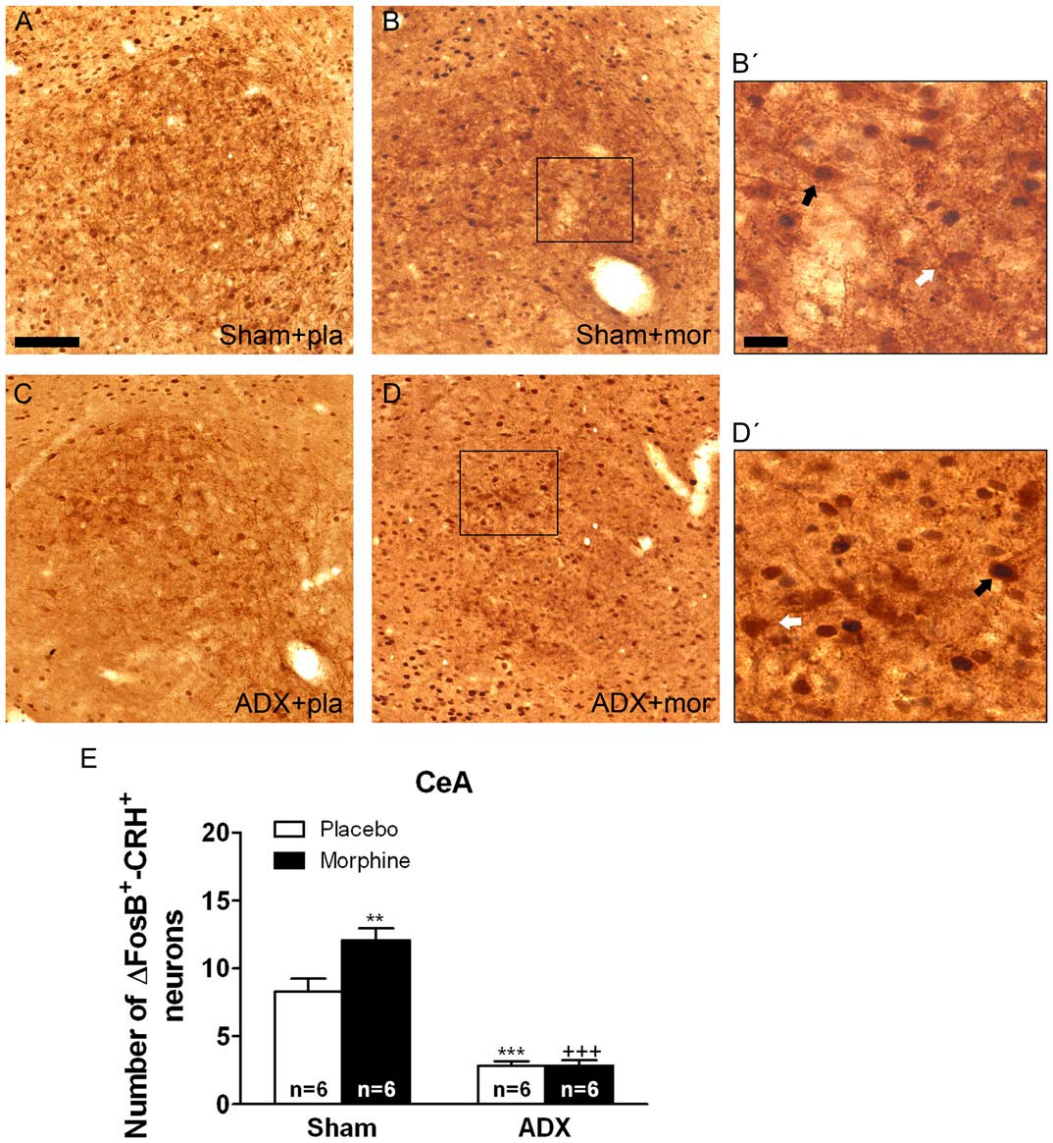
Adrenalectomy antagonized FosB/ΔFosB protein expression in the CeA CRH-positive neurons. ADX and non-operated (sham) rats were made dependent on morphine (mor) for 10 days. Controls received placebo pellets (pla). Animals were perfused and the CeA was processed for double-labelled (FosB/ΔFosB and CRH) immunohistochemistry. Panels A-D show immunohistochemical detection of FosB/ΔFosB into CRH neurons after different treatments. Low and high magnifications (B’, D’) images show FosB/ΔFosB-positive (blue-black)/CRH-positive (brown) neurons (black arrow) and FosB/ΔFosB-negative/CRH-positive (white arrow) immunoreactivity. Scale bar: 100 µm (80X, low magnification); 20 µm (275X, high magnification). E: quantitative analysis of FosB/ΔFosB-positive/CRH-positive neurons in the CeA. Data correspond to mean ± SEM. *Post hoc* test revealed a significant higher number of FosB/ΔFosB-positive nuclei into CRH immunoreactive neurons in morphine-dependent rats (**p<0.01 versus sham+placebo). ADX attenuated the increase of FosB/ΔFosB expression in CRH-positive neurons both in placebo- (***p<0.001 versus sham+placebo) and in morphine-treated animals (^+++^p<0.001 versus sham+morphine).

Two-way ANOVA for FosB/ΔFosB-positive/CRH-positive neurons in the BNST showed main effect of adrenalectomy [F(1,20) = 64.43, p*<*0.0001] and significant interaction between adrenalectomy and morphine treatment [F(1,20) = 6.80, p* = *0.0169]. In the PVN, two-way ANOVA revealed significant main effect of adrenalectomy [F(1,19) = 11.35, p* = *0.0032]. Two-way ANOVA for FosB/ΔFosB-positive/CRH-positive neurons in the CeA showed a significant main effect of adrenalectomy [F(1,20) = 106.85, p*<*0.0001], morphine treatment [F(1,20) = 7.33, p* = *0.0136] and significant interaction between adrenalectomy and drug pretreatment [F(1,20) = 7.07, p* = *0.0151]. Figures 4, 5, 6 show representative images of BNST, PVN and CeA sections from sham or ADX-controls and morphine-dependent rats. *Post hoc* analysis revealed that a significant number of FosB/ΔFosB-positive neurons were found to co express CRH in the BNST (p*<*0.01; Fig. 4E), PVN (p*<*0.05; Fig. 6E) and CeA (p*<*0.01; Fig. 6E) in sham-morphine rats compared with sham-placebo group. Additionally, the number of FosB/ΔFosB-positive neurons co expressing CRH in ADX placebo-pelleted rats and in morphine-dependent rats was significantly lower in the three nuclei when compared with the corresponding treatment in sham animals, as shown in Figures 4, 5, 6 (BNST: p*<*0.01 vs. sham plus placebo; p*<*0.001 vs. sham plus morphine; PVN: p*<*0.05 vs. sham plus placebo; p*<*0.001 vs. sham plus morphine; CeA: p*<*0.001 vs. sham plus placebo and vs. sham plus morphine).

At the BNST level, two-way ANOVA for the number of CRH-positive neurons showed that there was a main effect of ADX [F(1,20) = 103.92, p<0.0001] as well as chronic morphine [F(1,20) = 4.35, p<0.05]. As shown in Table 1, Newman–Keuls *post hoc* test revealed that ADX placebo- and morphine-pelleted rats showed lower CRH neurons (p<0.001) than the sham-placebo or morphine-dependent groups. At the PVN, two-way ANOVA showed a significant effect of adrenalectomy [F(1,19) = 11.35, p = 0.0032]. Two-way ANOVA for total CRH neurons at the CeA level revealed significant effects of ADX [F(1,20) = 240.09, p<0.0001] and main interaction between ADX and chronic morphine [F(1,20) = 4.49, p = 0.0467]. Table 1 depicts that there was a significant decrease in CRH neurons at the CeA from ADX placebo- or morphine-treated rats. Table 1 also shows that chronic morphine exposure induces an elevation (p<0.05) of CRH-positive neurons at BNST and CeA levels.

**Table 1:** Table **1.** Effects of adrenalectomy on the number of CRH-positive neurons in the BNST, PVN, and CeA, pro-DYN-positive neurons in the NAc and TH-positive neurons in the NTS, in placebo- or morphine-pelleted animals.

	BNST	PVN	CeA	NAc	NTS-A_2_
Treatment	CRH^+^	CRH^+^	CRH^+^	pro-Dyn^+^	TH^+^
Sham-placebo	21.05±1.62	66.29±3.22	24.41±0.85	22.42±1.31	26.03±2.87
Sham-morphine	27.50±2.70*	63.12±5.19	29.84±2.62*	25.31±1.64	25.31±0.97
ADX-placebo	6.09±0.87***	53.72±3.38	5.69±0.35***	18.55±0.95	21.21±1.53
ADX-morphine	6.91±1.18^+++^	49.58±3.06	5.19±0.30^+++^	22.95±1.98	20.18±0.91

Each value represents the mean ± SEM. n = 6 per group. In ADX rats, Newman–Keuls’ post hoc comparison test revealed a significant decrease in the number of CRH^+^ neurons in the BNST and CeA (*p<0.05; ***p<0.001, versus sham+placebo; ^+++^p<0.01, versus sham+morphine). CRH, corticotropin-releasing factor; PVN, hypothalamic paraventricular nucleus; BNST, bed nucleus of the stria terminalis; CeA, central nucleus amygdala; NAc, nucleus accumbens; NTS-A_2_ nucleus of the solitary tract-A_2_ noradrenergic cell group.

### Effects of Adrenalectomy on FosB/ΔFosB into DYN-positive Neurons in the NAc(Shell)

**Figure 7:**
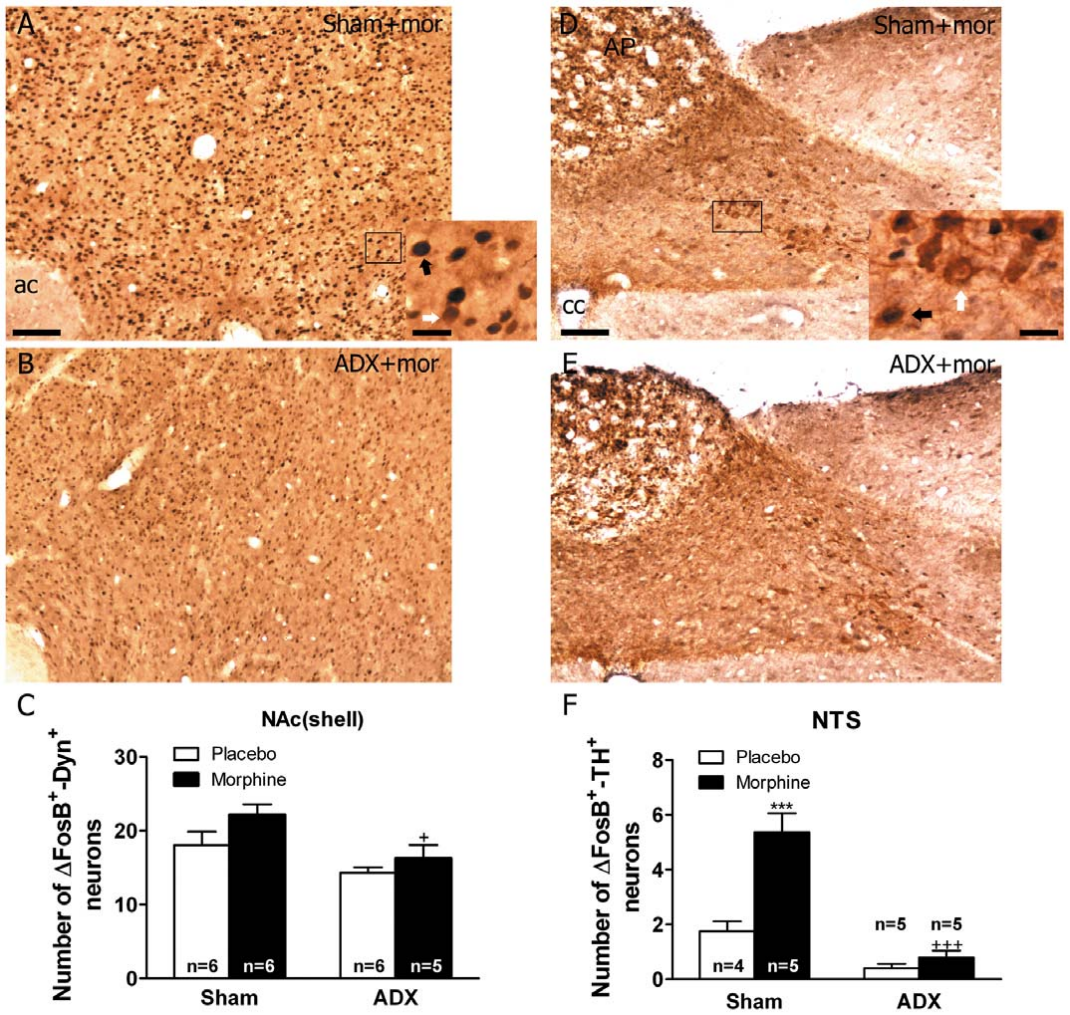
Effects of adrenalectomy on FosB/ΔFosB protein expression in the NAc(shell) pro-DYN-positive neurons and in the NTS TH-positive neurons from morphine dependent rats. ADX and non-operated (sham) rats were made dependent on morphine (mor) for 10 days. Controls received placebo pellets. Animals were perfused and the NAc and NTS were processed for double-labelled (FosB/ΔFosB and pro-DYN and FosB/ΔFosB and TH, respectively) immunohistochemistry. Panels A-B show immunohistochemical detection of FosB/ΔFosB into pro-DYN-positive neurons in sham- and ADX-morphine-dependent rats. Low and high magnifications images show FosB/ΔFosB-positive (blue-black)/pro-DYN-positive (brown) neurons (black arrow) and FosB/ΔFosB-negative/pro-DYN-positive (white arrow) immunoreactivity. Panels D-E show immunohistochemical detection of FosB/ΔFosB within TH-positive neurons in sham- and ADX-morphine-dependent rats. Low and high magnifications images show FosB/ΔFosB-positive (blue-black)/TH-positive (brown) neurons (black arrow) and FosB/ΔFosB-negative/TH-positive (white arrow) immunoreactivity. Scale bars: 100 µm (70X, low magnification); 20 µm (300X, high magnification). C: quantitative analysis of FosB/ΔFosB-positive/pro-DYN-positive neurons in the NAc(shell). F: quantitative analysis of FosB/ΔFosB-positive/TH-positive neurons in the NTS. Data correspond to mean ± SEM. ^+^p<0.05 versus sham+morphine. ac, anterior comisure, AP, area postrema; cc, canal central. Data correspond to mean ± SEM. ADX significantly attenuated the increase of FosB/ΔFosB expression in pro-DYN-positive neuron in the NAc and in TH-positive neurons in the NTS from morphine-dependent animals (***p<0.001 versus sham+placebo; ^+^p<0.05,^ +++^p<0.001 versus sham+morphine).

We observed no significant changes in the number of pro-DYN-positive cells that co-express FosB/ΔFosB in the NAc(shell) after chronic morphine exposure (Fig. 7) regarding to the placebo group. Two-way ANOVA for FosB/ΔFosB-positive/pro-DYN-positive neurons in the NAc(shell) showed main effect of ADX [F(1,19) = 10.11, p* = *0.0049]. As shown in Fig. 7C, the number of FosB/ΔFosB-positive neurons that co-express pro-DYN in ADX morphine-dependent rats was significantly (p*<*0.05) lower regarding to the corresponding treatment in sham animals. As shown in Table 1, ADX induced no changes in total pro-DYN cell from the NAc(shell).

### Adrenalectomy Inhibits Morphine Dependence-induced FosB/ΔFosB into TH-Positive Neurons in the NTS-A_2_ Noradrenergic Cell Group

Two-way ANOVA for FosB/ΔFosB-positive/TH-positive neurons in the NTS-A_2_ showed main effect of ADX [F(1,15) = 64.86, p*<*0.0001], morphine treatment [F(1,15) = 29.62, p*<*0.0001], and significant interaction between adrenalectomy and morphine treatment [F(1,15) = 19.24, p* = *0.0005]. Newman-Keuls *post hoc* test indicates that a significant number of FosB/ΔFosB-positive neurons were found to co-express TH in the NTS (p*<*0.001; Fig. 7F) in sham-morphine rats. In addition, the number of FosB/ΔFosB-positive neurons co-expressing TH in ADX morphine-dependent rats was significantly lower (p*<*0.001) when compared with the corresponding treatment in sham animals, as shown in Fig. 7F. On the other hand, as shown in Table 1, ADX induced no changes in total TH-positive neurons in the NTS.

## Discussion

The current results indicated, for the first time, that brain GC signalling modulated chronic morphine administration-induced FosB/ΔFosB expression in the brain stress system in a region-specific manner.

Converged lines of evidence indicate that stress increases risk of addictive behaviours [18]. Persistent stressful stimuli alter synthesis, expression and signalling in stress-related pathways (e.g. CRH, GC, NA, etc). In addition, drugs of abuse affect the stress pathways, which results in alteration of in gene expression, with signalling effects on reward and stress-related molecules [31]. Stress and abused drugs share the ability to trigger overlapping patterns of neuronal activation within the central nervous system, resulting in the activation of immediate gene expression. It has been extensively described that addictive substances and chronic stressful stimuli increase the expression of the transcription factor ΔFosB in the main nuclei involved in their positive reinforcing effects [12], [13], [32], [33], and it has been proposed that the persistent effects of ΔFosB on target genes might play an important role in the development of adaptations that characterize addiction [9], [34]. However, little is known about either the expression of ΔFosB in the brain stress system after chronic administration of drugs of abuse or the molecular mechanisms of morphine-evoked ΔFosB accumulation in stress-related areas. In the present study, we investigated the involvement of GC on the morphine-induced FosB/ΔFosB expression in the hormonal stress system (HPA axis), which is controlled by CRH in the PVN, as well as in the extrahypothalamic stress systems (which include the extended amygdala; [35]), mediated by CRH and other stress-related systems (including NA, and dynorphin; [19]).

We recently demonstrated that chronic morphine administration for seven days increased FosB/ΔFosB expression in the extended amygdala, PVN and NTS-A_2_ [14]. Consistent with these data, present results show that chronic morphine administration evoked an increase in FosB/ΔFosB in CeA, BNST and NAc(shell) as well as in the PVN and NTS-A_2_. The extended amygdala has been associated with drug reward. In fact, all major drugs of abuse activate dopaminergic transmission from the VTA to the NAc(shell) and CeA. This activation and the consequent positive reinforcing effects of substances of abuse have been shown to be related with GC actions on GR located in the VTA [28]. Supporting this hypothesis, our results show that FosB/ΔFosB-IR in the NAc(shell) and the CeA was attenuated in ADX animals, which might indicate a cross-talk between AP-1 transcription factors and GR during morphine dependence [36]. In contrast to the observed effects of ADX on FosB expression in NAc and CeA, present results suggest that chronic morphine increased Fos/ΔFosB expression in the PVN and BNST in a GC-independent manner.

Several neurotransmitters of the brain stress system, such as CRH, NA and DYN, have been related with the aversive states characteristic of the addiction process [35], [37]–[39]. Present findings showed that chronic morphine exposure evoked a significant increase in Fos/ΔFosB expression within CRH-containing neurons in the BNST, CeA, and PVN. The Fos family of transcription factors can act at cyclic AMP-response element (CRE) sites [40]. Considerable evidence indicates that ΔFosB can act as either a transcriptional repressor or activator [40], [41]. Given that CRH gene has a CRE motif in its promoter sequence, it might be proposed that FosB/ΔFosB accumulation in CRH neurons can mediate the morphine-induced changes in CRH levels, as reported for cocaine effects [42], especially in the CeA and the BNST, where, for the first time, we reported an enhancement in the number of CRH-positive neurons during morphine dependence. Supporting this hypothesis, an increase in CRH mRNA levels has been described in the CeA after chronic administration of morphine [43]. However, the number of CRH-positive neurons was unchanged in the PVN after chronic morphine treatment. These results are in accordance with previous findings showing that chronic morphine exposure does not induce changes in the PVN CRH hnRNA [44]. Since PVN CRH expression is down-regulated by GC [45], and given that present work and others [29], [44] showed that chronic opiate exposure did not modify corticosterone release, it seems logical that chronic morphine administration did not change the number of CRH cells.

Peripheral administration of corticosterone increases CRH mRNA expression in the CeA and BNST [46], [47]. Additionally, ADX decreased CRH expression in the CeA [48], [49]. Accordingly, we have observed that the increase in the number of CRH neurons in the BNST and CeA during morphine dependence was abolished after adrenalectomy. Given that the increase of Fos/ΔFosB within CRH neurons during morphine dependence was attenuated in ADX animals, it might be suggested a role for Fos/ΔFosB in the regulation of CRH expression by GC in the extended amygdala. On the other hand, it is well known that GC negatively regulate the expression of CRH gene in the PVN [45]. In agreement, present data clearly showed that adrenalectomy abolished the increase in Fos/ΔFosB-IR in CRH-containing neurons during morphine dependence in this nucleus.

In this study, immunochemical data revealed that chronic morphine treatment significantly increased staining for Fos/ΔFosB in the NTS-A_2_, which was decreased following ADX. When we examined the specific neural populations that expressed FosB/ΔFosB, we found a robust increase of Fos/ΔFosB-IR within TH (the rate-limiting enzyme in catecholamine synthesis)-positive neurons. NA has been shown to play a main role in addiction [50]. In prior work, chronic morphine exposure evoked an enhancement in TH levels in the NTS-A_2_ [14], [29]. It is known that TH gene has an AP-1 site in its promoter [51]. Our results might suggest that FosB/ΔFosB is involved in opiate-induced increase of TH levels in the NTS-A_2_. Brainstem noradrenergic cell group express high levels of GC receptors (GR). It has been shown that GC have a permissive role in noradrenergic neurotransmission [29], [30], [52]. Accordingly, administration of GR antagonists affected several aspects of NA activity, including TH activation and neuronal activity [30]. Present results showed that, following ADX there was a decrease in TH-positive cells expressing FosB/ΔFosB in the NTS-A_2_. Taken into account that adrenalectomy blocked the increase in TH protein levels in the NTS-A_2_ during morphine dependence [29], and that a GRE/AP-1 site has been described in the TH gene promoter [53], our data might point out ΔFosB as a mediator in the effects of GC on noradrenergic activity in the NTS-A_2_ during opiate dependence.

DYN has been postulated as a possible target gene of ΔFosB [54], [55]. Our results show that chronic morphine exposure did not significantly altered FosB/ΔFosB expression within pro-DYN-expressing neurons in the NAc(shell). Regarding ΔFosB regulation of DYN expression, Zachariou et al [33] reported a small but significant decrease of DYN mRNA levels in the NAc from ΔFosB over-expressing mice, thus postulating that this transcription factor inhibits DYN expression. From our data, it cannot be concluded that chronic morphine modifies DYN expression through FosB/ΔFosB activity, given that our results have been obtained at the protein level.

It has been postulated that DYN/kappa opioid system seems to induce pro-depressive-like states that involve elements of aversion. This aversive response may involve reciprocal interactions with NAc, DA and the extrahypothalamic CRH system [35]. However, little is known about a possible GC regulation of DYN expression in the NAc. It has been proposed that ΔFosB in the NAc, partly through the repression of DYN expression, increases the sensitivity to the rewarding effects of morphine and cocaine and leads to resilience to stress [9], [33]. Our data support a role for GC in regulating the positive reinforcing effects of morphine mediated by the mesocorticolimbic DA system, given that the number of FosB/ΔFosB/pro-DYN-positive neurons decrease in the NAc from ADX rats. These effects could be mediated directly by GR, which are present throughout the mesolimbic reward pathway, or indirectly via CRH projections arising from the CeA and/or the BNST to the VTA and NAc, which are underactivated during morphine dependence in ADX rats.

In summary, this study provides evidence that GC are critically involved in FosB/ΔFosB accumulation in the brain stress systems after chronic morphine exposure, which might result in lasting changes of gene expression pattern in stress-related areas. The present findings also indicate that FosB/ΔFosB may contribute to the GC-dependent changes on brain stress system plasticity during opiate dependence. Further studies are necessary to determine the intracellular mechanisms by which chronic opiates induce FosB/ΔFosB in selected stress-related regions, as well as the mechanism that is responsible for the suppression of morphine-induced FosB/ΔFosB expression by GC.

## Methods

### Materials

Corticosterone and cholesterol were purchased from Sigma Chemical Co. (St Louis, MO, USA). Pellets of corticosterone were made by Dr. Márton Vajna, (Department of Pharmacy Administration, University of Pharmacy, Semmelweis University, Budapest, Hungary). Pellets of morphine base (Alcaliber Laboratories, Madrid, Spain) or lactose (control) were prepared in the Department of Pharmacy and Pharmaceutics Technology (School of Pharmacy, Granada, Spain). Pentobarbital was purchased from Hospira (Hoofddorp, The Netherlands). Ketamine chlorhydrate and xylazine were purchased from Labs. Merial (Lyon, France) and Labs. Calier (Barcelona, Spain), respectively.

### Animals

Male Sprague-Dawley rats (220–240 g at the beginning of the experiment; Harlan, Barcelona, Spain) were housed in pairs in cages (length, 45 cm; width, 24 cm; height, 20 cm) on arrival in a room with controlled temperature (22±2°C) and humidity (50±10%), with free access to water and food (Harlan Teklad standard rodent chow; Harlan Interfauna Ibérica, Barcelona, Spain). Animals were adapted to a standard 12 h light-dark cycle (lights on: 08 h 00 min –20 h 00 min) for 7 days before the beginning of the experiments. All surgical and experimental procedures were performed in accordance with the European Communities Council Directive of 24 November 1986 (86/609/EEC), and were approved by the local Committees for animal research (REGA ES300305440012). The study was approved by the University of Murcia bioethics committee (RD 1201/2005) and Ministerio de Ciencia y Tecnología (SAF/FEDER 2009-07178), Spain.

### Adrenalectomy

Rats were bilaterally adrenalectomized and a corticosterone pellet was implanted to ensure low but stable levels of GC [29]. Rats were bilaterally adrenalectomized (ADX) via a dorsal approach under 90 mg/kg ketamine chlorhydrate and 8 mg/kg xylazin (i.p.) anaesthesia, and implanted subcutaneously (s.c.) with slow-release corticosterone pellets at surgery. The composition of steroid pellets (25 mg corticosterone plus 75 mg cholesterol) was chosen to provide stable corticosterone concentration corresponding to circadian nadir up to 20 d after implantation [56]. ADX rats with corticosterone replacement (ADX plus corticosterone) do not mount a challenge-induced increase of plasma corticosterone [56]. After surgery, ADX plus corticosterone rats had free choice to drink isotonic saline (0.9% NaCl) to replace depleted sodium secondary to the loss of aldosterone because of adrenalectomy. Control rats were subjected to the same surgical procedure (sham) without adrenal extirpation. Sham and ADX plus corticosterone rats were allowed to recover from surgery for 5 d before the morphine-dependence procedure. Successful bilateral adrenalectomy was confirmed by plasma concentration of corticosterone and ACTH and by post-mortem examination of the ADX animals.

### Drug Treatment and Experimental Procedure

Five days after surgery, rats were implanted s.c. with two 75 mg morphine pellets under light ether anaesthesia. Control rats received placebo pellets containing lactose. This procedure has been shown to produce consistent plasma morphine concentrations beginning a few hours after the implantation of the pellets and a full withdrawal syndrome after acute injection of opioid antagonists [57]. Dependence on morphine is achieved 24 h after implantation of pellets and remained constant for 15 days [58]. Ten days after the implantation of morphine or placebo pellets, rats were sacrificed. The four experimental conditions investigated for HPA axis activity (plasma concentration of corticosterone and ACTH), FosB/ΔFosB expression, CRH expression, DYN expression, TH expression, and FosB/ΔFosB expression into CRH-, DYN- and TH-positive neurons were: (i) sham-placebo; (ii) sham-morphine; (iii) ADX-placebo; (iv) ADX-morphine. The weight gain of the rats was checked during treatment to ensure that the morphine was liberated correctly from the pellets, because it is known that long-term morphine treatment induces a decrease in body weight gain caused by lower caloric intake [29].

### Brain Perfusion and Sectioning

Rats were deeply anaesthetised with an overdose of pentobarbital (100 mg/kg i.p.) and perfused transcardially with saline following by fixative containing paraformaldehyde (4% paraformaldehyde in 0.1 M borate buffer, pH 9.5). After removal of the perfused brains, they were post fixed in the same fixative for 3 h and stored at 4°C in PBS containing 10% sucrose until coronal sections (30-µm thickness) were cut rostrocaudally on a freezing microtome (Leica, Nussloch, Germany). The atlas of Paxinos and Watson (2007) [59] was used to identify different brain regions of rats: NAc(shell), BNST, PVN, CeA and NTS-A_2_ (Figure 1). The sections were cryoprotected and stored at −20°C until use.

### FosB/ΔFosB Immunohistochemistry

Sections were processed for immunohistochemistry as described by Núñez et al [29]. Briefly, after blocking with 0.3% H_2_O_2_ and 2% normal goat serum (Sigma, USA) tissue sections were incubated in primary anti-FosB/ΔFosB antiserum (rabbit polyclonal, #sc-48, Santa Cruz Biotechnology, Santa Cruz, CA, USA; 1∶1000). The primary antibody used in this study does not discriminate between FosB and its stable splice variant ΔFosB. Repeated exposure to FosB-inducing stimuli has been shown to desensitise acute FosB inductibility [15], [40], [60] . Therefore, differences between FosB/ΔFosB staining in morphine-pretreated and non-pretreated rats can be interpreted as primarily reflecting differences in ΔFosB accumulation. Antigens were visualised by conventional avidin-biotin-immunoperoxidase protocol (Vectastain ABC Elite Kit; Vector Laboratories, Burlingame, CA, USA). 3,3′-diaminobenzidine tetrahydrochloride (DAB; Sigma, USA) reaction was intensified with nickel-ammonium sulphate. Sections were mounted onto chrome-alum gelatine-coated slides, dehydrated and coverslipped.

### Double-labelling Immunohistochemistry of FosB/ΔFosB-immunoreactive Nuclei and CRH-, TH- and DYN-positive Neurons

For FosB/ΔFosB-TH double-labelling, tissue sections from each rat in each treatment group were processed for FosB/ΔFosB immunoreactivity using DAB nickel intensification and then TH was revealed using DAB chromogen only. FosB/ΔFosB immunostaining was performed as described previously. Following the FosB/ΔFosB staining, sections were rinsed in PBS, treated with 2% normal goat serum and then incubated overnight with the rabbit polyclonal anti-TH antibody (AB152, Chemicon, USA; 1∶6000) at room temperature. The same immunohistochemistry procedures described above were followed. The TH antibody–peroxidase complex was developed in DAB. The sections were mounted onto chrome-alum gelatine coated slides and coverslipped.

For the FosB/ΔFosB-CRH and FosB/ΔFosB-pro-DYN double labelling, the process was the same as described before for FosB/ΔFosB-TH. For detecting DYN-expressing neurons in the NAc, an antigen retrieval procedure was applied by incubating brain sections in citrate buffer (10 mM Citric Acid, 0.05% Tween 20, pH 6.0) at 60°C for 20 min before the blocking procedure. The primary anti-CRH rabbit antiserum was kindly provided by Wylie W. Vale (The Salk Institute, La Jolla, CA, USA), and was used at 1∶500 dilution for 72 h at 4°C. The pro-DYN primary antibody was purchased from Neuromics (# GP10110, Neuromics, Edina, MN, USA) and diluted 1∶2000 (72 h, 4°C).

### Image Analysis

Images were captured by means of Leica microscope (DM 4000B; Leica) connected to a video camera (DFC290, Leica). FosB/ΔFosB-positive cell nuclei were counted using a computer-assisted image analysis system (QWIN, Leica). The boundaries of the NTS-A_2_, BNST, CeA, NAc(shell) and the parvocellular subdivision of the PVN were outlined and the number of positive profiles was recorded. The number of FosB/ΔFosB nuclear profiles within the confines of cell groups of interest was counted bilaterally in four to five sections from each rat and averaged to obtain a single value for each rat. To avoid observer bias, all sections were quantified by a blinded investigator. Total counts for different brain regions are expressed as mean ± SEM.

### Quantification of TH-, CRH- and DYN-positive Cells and FosB/ΔFosB Double Stained Profiles

Positive nuclei for FosB/ΔFosB immunoreactivity were detected using the same conventional light microscopy described above, and counted at x40 magnification. FosB/ΔFosB–positive CRH, TH or DYN cells were identified as cells with brown cytosolic deposits for CRH-positive, TH-positive and DYN-positive staining and blue/dark nuclear staining for FosB/ΔFosB. A square field (195 µm) was superimposed upon captured image to use as reference area. The number of double-labelled neurons observed bilaterally was counted in four to five sections from each animal in the PVN, BNST and CeA for CRH neurons, NTS for TH neurons and NAc for DYN neurons. The CRH, TH and DYN positive cells without a visible nucleus (FosB/ΔFosB-negative CRH, TH or DYN cells) were also included in the analysis.

### Radioimmunoassay

Blood was collected into ice-cooled tubes containing 5% EDTA and was then centrifuged (500 *g*; 4°C; 15 min). Plasma was separated, divided into two aliquots and stored at −80°C until assayed for corticosterone or ACTH. Plasma concentration of corticosterone and ACTH were quantified using specific corticosterone and ACTH antibodies for rats ([^125^I]-CORT and [^125^I]-ACTH RIA; MP Biomedicals, Orangeburg, NY, USA). The sensitivity of the assay was 7.7 ng/mL for corticosterone and 5.7 pg/mL for ACTH.

### Statistical Analysis

Data are presented as mean ± SEM and were analyzed using the GraphPad Prism statistical package (San Diego, CA, USA). Data were analysed by two-way analysis of variance (ANOVA) with treatment (placebo, morphine) and surgery (sham, ADX) as independent variables. The Newman–Keuls *post hoc* test was used for individual group comparisons. Differences with a p<0.05 were considered significant.

## Supporting Information

Figure S1 **Effects of adrenalectomy (ADX) on plasma ACTH (A) and corticosterone (B) concentrations in controls and in morphine-treated animals.** Surgical ADX increased ACTH levels both in placebo- and in morphine-treated rats, whereas decreased plasma corticosterone concentration in morphine-treated rats. Data represent the mean ± SEM of plasma ACTH and corticosterone levels in rats pretreated with placebo or morphine for 10 days. ***p*<*0.001 versus sham-placebo; ^++^p*<*0.01, ^+++^p*<*0.001 versus sham-morphine.
(TIF)

## References

[1] Volkow ND, Skolnick P (2012) New Medications for Substance Use Disorders: Challenges and Opportunities. Neuropsychopharmacology 37: 290–292. 22157859 10.1038/npp.2011.84 PMC3238092

[2] Robison AJ, Nestler EJ (2011) Transcriptional and epigenetic mechanisms of addiction. Nat Rev Neurosci 12: 623–637. 21989194 10.1038/nrn3111 PMC3272277

[3] Hutchison ER, Okun E, Mattson MP (2009) The Therapeutic Potential of microRNAs in Nervous System Damage, Degeneration and Repair. Neuromolecular Med 11: 153–161. 19763905 10.1007/s12017-009-8086-x PMC2757407

[4] Li MD, van der Vaart AD (2011) MicroRNAs in addiction: adaptation’s middlemen? Mol Psychiatry 16: 1159–1168. 21606928 10.1038/mp.2011.58 PMC4251867

[5] Mumberg D, Lucibello FC, Schuermann M, Muller R (1991) Alternative splicing of fosB transcripts results in differentially expressed mRNAs encoding functionally antagonistic proteins. Genes Dev 5: 1212–1223. 1648531 10.1101/gad.5.7.1212

[6] Chen J, Kelz MB, Hope BT, Nakabeppu Y, Nestler EJ (1997) Chronic Fos-Related Antigens: Stable Variants of ΔFosB Induced in Brain by Chronic Treatments. J Neurosci 17: 4933–4941. 9185531 10.1523/JNEUROSCI.17-13-04933.1997 PMC6573301

[7] Moratalla R, Elibol B, Vallejo M, Graybiel AM (1996) Network-Level Changes in Expression of Inducible Fos-Jun Proteins in the Striatum during Chronic Cocaine Treatment and Withdrawal. Neuron 17: 147–156. 8755486 10.1016/s0896-6273(00)80288-3

[8] Chao J, Nestler EJ (2004) Molecular Neurobiology of Drug Addiction. Annual Review of Medicine 55: 113–132. 10.1146/annurev.med.55.091902.103730 14746512

[9] Muschamp JW, Nemeth CL, Robison AJ, Nestler EJ, Carlezon J (2012) ΔFosB Enhances the Rewarding Effects of Cocaine While Reducing the Pro-Depressive Effects of the Kappa-Opioid Receptor Agonist U50488. Biological Psychiatry 71: 44–50. 21962331 10.1016/j.biopsych.2011.08.011 PMC3230776

[10] Gago B, Suárez-Boomgaard D, Fuxe K, Brené S, Reina-Sánchez MD, et al (2011) Effect of acute and continuous morphine treatment on transcription factor expression in subregions of the rat caudate putamen. Marked modulation by D4 receptor activation. Brain Res 1407: 47–61. 21782156 10.1016/j.brainres.2011.06.046

[11] Kaplan GB, Leite-Morris KA, Fan WY, Young AJ, Guy MD (2011) Opiate Sensitization Induces FosB/ΔFosB Expression in Prefrontal Cortical, Striatal and Amygdala Brain Regions. PLoS ONE 6: e23574. 21886798 10.1371/journal.pone.0023574 PMC3160315

[12] Nye HE, Nestler EJ (1996) Induction of chronic fos-related antigens in rat brain by chronic morphine administration. Mol Pharmacol 49: 636–645. 8609891

[13] McClung CA, Nestler EJ, Zachariou V (2005) Regulation of Gene Expression by Chronic Morphine and Morphine Withdrawal in the Locus Ceruleus and Ventral Tegmental Area. J Neurosci 25: 6005–6015. 15976090 10.1523/JNEUROSCI.0062-05.2005 PMC6724795

[14] Núñez C, Martín F, Földes A, Laorden ML, Kovács KJ, et al (2010) Induction of FosB/ΔFosB in the brain stress system-related structures during morphine dependence and withdrawal. Journal of Neurochemistry 114: 475–487. 20438612 10.1111/j.1471-4159.2010.06765.x

[15] Perrotti LI, Hadeishi Y, Ulery PG, Barrot M, Monteggia L, et al (2004) Induction of ΔFosB in Reward-Related Brain Structures after Chronic Stress. J Neurosci 24: 10594–10602. 15564575 10.1523/JNEUROSCI.2542-04.2004 PMC6730117

[16] Vialou V, Robison AJ, LaPlant QC, Covington HE, Dietz DM, et al (2010) ΔFosB in brain reward circuits mediates resilience to stress and antidepressant responses. Nat Neurosci 13: 745–752. 20473292 10.1038/nn.2551 PMC2895556

[17] Ambroggi FC, Turiault M, Milet A, Deroche-Gamonet V, Parnaudeau S, et al (2009) Stress and addiction: glucocorticoid receptor in dopaminoceptive neurons facilitates cocaine seeking. Nat Neurosci 12: 247–249. 19234455 10.1038/nn.2282

[18] Sinha R (2008) Chronic stress, drug use, and vulnerability to addiction. Ann N Y Acad Sci 1141: 105–130. 18991954 10.1196/annals.1441.030 PMC2732004

[19] Koob GF, Volkow ND (2010) Neurocircuitry of addiction. Neuropsychopharmacology 35: 217–238. 19710631 10.1038/npp.2009.110 PMC2805560

[20] Koob G, Kreek MJ (2007) Stress, Dysregulation of Drug Reward Pathways, and the Transition to Drug Dependence. Am J Psychiatry 164: 1149–1159. 17671276 10.1176/appi.ajp.2007.05030503 PMC2837343

[21] Koob GF, Le Moal M (2008) Neurobiological mechanisms for opponent motivational processes in addiction. Phil Trans R Soc B 363: 3113–3123. 18653439 10.1098/rstb.2008.0094 PMC2607326

[22] Koob GF (2006) The neurobiology of addiction: a neuroadaptational view relevant for diagnosis. Addiction 101: 23–30. 16930158 10.1111/j.1360-0443.2006.01586.x

[23] Carrasco GA, Van de Kar LD (2003) Neuroendocrine pharmacology of stress. European Journal of Pharmacology 463: 235–272. 12600714 10.1016/s0014-2999(03)01285-8

[24] Piazza PV, Le Moal M (1997) Glucocorticoids as a biological substrate of reward: physiological and pathophysiological implications. Brain Research Reviews 25: 359–372. 9495563 10.1016/s0165-0173(97)00025-8

[25] Cleck JN, Blendy JA (2008) Making a bad thing worse: adverse effects of stress on drug addiction. J Clin Invest 118: 454–461. 18246196 10.1172/JCI33946 PMC2214707

[26] Goodman A (2008) Neurobiology of addiction. An integrated review. Biochem Pharmacol 75: 266–322. 17764663 10.1016/j.bcp.2007.07.030

[27] Dunn AJ, Swiergiel AH (2008) The role of corticotropin-releasing factor and noradrenaline in stress-related responses, and the inter-relationships between the two systems. European Journal of Pharmacology 583: 186–193. 18281033 10.1016/j.ejphar.2007.11.069 PMC2661014

[28] Koob GF, Le Moal M (2008) Addiction and the brain antireward system. Ann Rev Physiol 59: 29–53. 10.1146/annurev.psych.59.103006.093548 18154498

[29] Núñez C, Földes A, Pérez-Flores D, García-Borrón JC, Laorden ML, et al (2009) Elevated glucocorticoid levels are responsible for induction of tyrosine hydroxylase (TH) mRNA expression, phosphorylation and enzyme activity in the nucleus of the solitary tract (NTS-A2) during morphine withdrawal. Endocrinology 150: 3118–3127. 19179436 10.1210/en.2008-1732 PMC2703550

[30] Navarro-Zaragoza J, Hidalgo JM, Laorden ML, Milanés MV (2012) Glucocorticoid receptors participate in the opiate withdrawal-induced stimulation of rats NTS noradrenergic activity and in the somatic signs of morphine withdrawal. Br J Pharmacol DOI: 10.1111/j.1476–5381.2012.01918.x</P>. 10.1111/j.1476-5381.2012.01918.x PMC3402777 22364199

[31] Renthal W, Nestler EJ (2008) Epigenetic mechanisms in drug addiction. Trends in Molecular Medicine 14: 341–350. 18635399 10.1016/j.molmed.2008.06.004 PMC2753378

[32] Sim-Selley LJ, Cassidy MP, Sparta A, Zachariou V, Nestler EJ, et al (2011) Effect of ΔFosB overexpression on opioid and cannabinoid receptor-mediated signaling in the nucleus accumbens. Neuropharmacology 61: 1470–1476. 21907220 10.1016/j.neuropharm.2011.08.046 PMC3261795

[33] Zachariou V, Bolanos CA, Selley DE, Theobald D, Cassidy MP, et al (2006) An essential role for ΔFosB in the nucleus accumbens in morphine action. Nat Neurosci 9: 205–211. 16415864 10.1038/nn1636

[34] Hyman SE, Malenka RC (2001) Addiction and the brain: The neurobiology of compulsion and its persistence. Nat Rev Neurosci 2: 695–703. 11584307 10.1038/35094560

[35] Koob GF (2008) A role for Brain Stress System in addiction. Neuron 59: 11–34. 18614026 10.1016/j.neuron.2008.06.012 PMC2748830

[36] Deroche-Gamonet V, Sillaber I, Aouizerate B, Izawa R, Jaber M, et al (2003) The Glucocorticoid Receptor as a Potential Target to Reduce Cocaine Abuse. J Neurosci 23: 4785–4790. 12805318 10.1523/JNEUROSCI.23-11-04785.2003 PMC6740779

[37] Nestler EJ (2001) Molecular basis of long-term plasticity underlying addiction. Nat Rev Neurosci 2: 119–128. 11252991 10.1038/35053570

[38] Laorden ML, Ferenczi S, Pintér-Kübler B, González-Martín LL, Lasheras MC, et al (2012) Hypothalamic Orexin-A Neurons Are Involved In The Response Of The Brain Stress System To Morphine Withdrawal. PLoS ONE 7: e36871. 22590628 10.1371/journal.pone.0036871 PMC3348891

[39] Navarro-Zaragoza J, Núñez C, Ruiz-Medina J, Laorden ML, Valverde O, et al (2011) CRF(2) mediates the increased noradrenergic activity in the hypothalamic paraventricular nucleus and the negative state of morphine withdrawal in rats. Br J Pharmacol 162: 851–862. 20973778 10.1111/j.1476-5381.2010.01090.x PMC3042196

[40] McClung CA, Ulery PG, Perrotti LI, Zachariou V, Berton O, et al (2004) DFosB: a molecular switch for long-term adaptation in the brain. Molecular Brain Research 132: 146–154. 15582154 10.1016/j.molbrainres.2004.05.014

[41] Nestler EJ (2008) Transcriptional mechanisms of addiction: role of DFosB. Phil Trans R Soc B 363: 3245–3255. 18640924 10.1098/rstb.2008.0067 PMC2607320

[42] Crespo JA, Manzanares J, Oliva JM, Corchero J, Garcia-Lecumberri C, et al (2003) Extinction of cocaine self-administration produces alterations in corticotropin releasing factor gene expression in the paraventricular nucleus of the hypothalamus. Molecular Brain Research 117: 160–167. 14559150 10.1016/s0169-328x(03)00316-4

[43] Maj M, Turchan J, Smialowska M, Przewlocka B (2003) Morphine and cocaine influence on CRF biosynthesis in the rat central nucleus of amygdala. Neuropeptides 37: 105–110. 12747942 10.1016/s0143-4179(03)00021-0

[44] Núñez C, Foldes A, Laorden ML, Milanes MV, Kovács KJ (2007) Activation of stress-related hypothalamic neuropeptide gene expression during morphine withdrawal. J Neurochem 101: 1060–1071. 17286593 10.1111/j.1471-4159.2006.04421.x

[45] Swanson LW, Simmons DM (1989) Differential steroid hormone and neural influences on peptide mRNA levels in CRH cells of the paraventricular nucleus: a hybridization histochemical study in the rat. J Comp Neurol 285: 413–435. 2569487 10.1002/cne.902850402

[46] Makino S, Gold PW, Schulkin J (1994) Effects of corticosterone on CRH mRNA and content in the bed nucleus of the stria terminalis; comparison with the effects in the central nucleus of the amygdala and the paraventricular nucleus of the hypothalamus. Brain Res 657: 141–149. 7820612 10.1016/0006-8993(94)90961-x

[47] Makino S, Gold PW, Schulkin J (1994) Corticosterone effects on corticotropin-releasing hormone mRNA in the central nucleus of the amygdala and the parvocellular region of the paraventricular nucleus of the hypothalamus. Brain Res 640: 105–112. 8004437 10.1016/0006-8993(94)91862-7

[48] Viau V, Soriano L, Dallman MF (2001) Androgens Alter Corticotropin Releasing Hormone and Arginine Vasopressin mRNA Within Forebrain Sites Known to Regulate Activity in the Hypothalamic-Pituitary-Adrenal Axis. Journal of Neuroendocrinology 13: 442–452. 11328455 10.1046/j.1365-2826.2001.00653.x

[49] Palkovits M, Young WS, Kovács K, Tóth Z, Makara GB (1998) Alterations in corticotropin-releasing hormone gene expression of central amygdaloid neurons following long-term paraventricular lesions and adrenalectomy. Neuroscience 85: 135–147. 9607709 10.1016/s0306-4522(97)00621-0

[50] Smith RJ, Aston-Jones G (2008) Noradrenergic transmission in the extended amygdala: role in increased drug-seeking and relapse during protracted drug abstinence. Brain Struct Funct 213: 43–61. 18651175 10.1007/s00429-008-0191-3 PMC3632504

[51] Sabban EL, Hiremagalur B, Nankova B, Kvetnanský R (1995) Molecular biology of stress-elicited induction of catecholamine biosynthetic enzymes. Ann N Y Acad Sci 771: 327–338. 8597411 10.1111/j.1749-6632.1995.tb44692.x

[52] Roozendaal B, Okuda S, de Quervain DJF, McGaugh JL (2006) Glucocorticoids interact with emotion-induced noradrenergic activation in influencing different memory functions. Neuroscience 138: 901–910. 16310958 10.1016/j.neuroscience.2005.07.049

[53] Rani CSS, Elango N, Wang SS, Kobayashi K, Strong R (2009) Identification of an Activator Protein-1-Like Sequence as the Glucocorticoid Response Element in the Rat Tyrosine Hydroxylase Gene. Mol Pharmacol 75: 589–598. 19060113 10.1124/mol.108.051219 PMC2645927

[54] Hughes P, Dragunow M (1995) Induction of immediate-early genes and the control of neurotransmitter-regulated gene expression within the nervous system. Pharmacol Rev 47: 133–178. 7784478

[55] Kovacs GL, Telegdy G (1988) Hypothalamo-neurohypophyseal neuropeptides and experimental drug addiction. Brain Res Bull 20: 893–895. 2842008 10.1016/0361-9230(88)90107-4

[56] Kovács KJ, Földes A, Sawchenko PE (2000) Glucocorticoid negative feedback selectively targets vasopressin transcription in parvocellular neurosecretory neurons. J Neurosci 20: 3843–3852. 10804224 10.1523/JNEUROSCI.20-10-03843.2000 PMC6772698

[57] Frenois F, Cador M, Caille S, Stinus L, Le Moine C (2002) Neural correlates of the motivational and somatic components of naloxone-precipitated morphine withdrawal. Eur J Neurosci 16: 1377–1389. 12405997 10.1046/j.1460-9568.2002.02187.x

[58] Gold LH, Stinus L, Inturrisi CE, Koob GF (1994) Prolonged tolerance, dependence and abstinence following subcutaneous morphine pellets implantation in the rat. Eur J Pharmacol 253: 45–51. 8013548 10.1016/0014-2999(94)90755-2

[59] Paxinos G. and Watson C (2007) The rat brain in stereotaxic coordinates. Amsterdam: Academic Press.

[60] Perrotti LI, Weaver RR, Robison B, Renthal W, Maze I, et al (2008) Distinct patterns of FosB induction in brain by drugs of abuse. Synapse 62: 358–369. 18293355 10.1002/syn.20500 PMC2667282

