# Supplementary figures and images for: Glucocorticoids Regulation of FosB/ΔFosB Expression Induced by Chronic Opiate Exposure in the Brain Stress System
Source: PLoS One. 2012 Nov 21;7(11):e50264. doi: 10.1371/journal.pone.0050264 (PMC3503985; doi:10.1371/journal.pone.0050264)

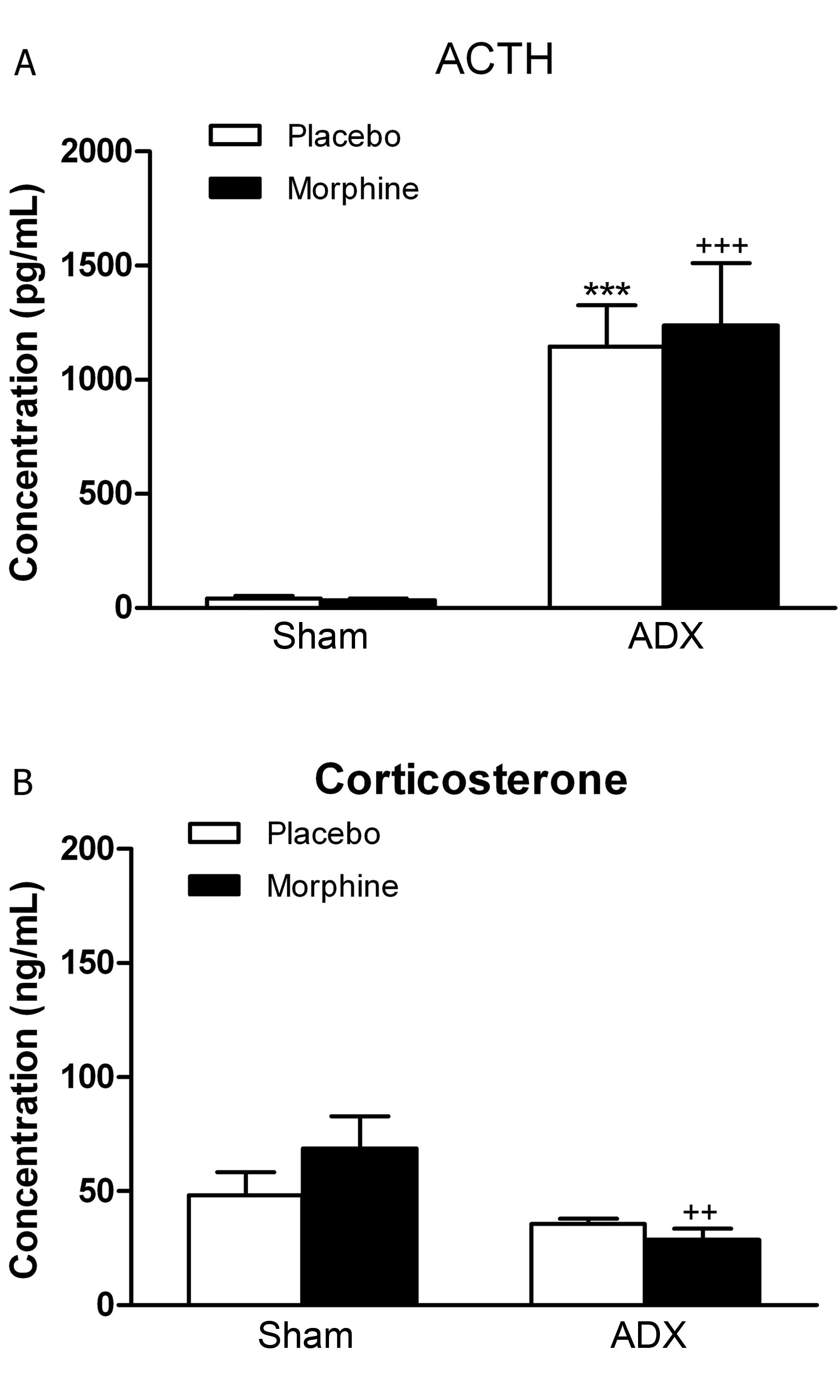

Supplement: Figure S1 — Effects of adrenalectomy (ADX) on plasma ACTH (A) and corticosterone (B) concentrations in controls and in morphine-treated animals. Surgical ADX increased ACTH levels both in placebo- and in morphine-treated rats, whereas decreased plasma corticosterone concentration in morphine-treated rats. Data represent the mean ± SEM of plasma ACTH and corticosterone levels in rats pretreated with placebo or morphine for 10 days. ***p<0.001 versus sham-placebo; ++p<0.01, +++p<0.001 versus sham-morphine. (TIF) [file pone.0050264.s001.tif]
